# Influence of continuous 4C nursing on quality of life and self-care ability of patients with diabetes retinopathy: An observational study

**DOI:** 10.1097/MD.0000000000037920

**Published:** 2024-05-17

**Authors:** Ayixianmuguli Wufuer, Jiamei Ma, Pazilaiti Ainiwa, Qi Zhou

**Affiliations:** a Ophthalmology Department of Xinjiang Uygur Autonomous Region People’s Hospital Urumqi, Xinjiang, China.

**Keywords:** compliance, continuous 4C care, diabetes retinopathy, quality of life, visual acuity level

## Abstract

This study aims to investigate the impact of continuous 4C nursing on patients with diabetes retinopathy (DR) and its influence on self-care ability. A total of 100 DR patients admitted to our hospital from October 2020 to October 2022 were randomly divided into a control group and an observation group, with 50 cases in each group. The control group received routine care, while the observation group received continuous 4C care. The nursing effects of both groups were compared. After nursing, the observation group showed a lower self-rating anxiety scale score and a higher self-care ability scale score compared to the control group (*P* < .05). The SQQL-VI scores for all social activities were also higher in the observation group (*P* < .05). Additionally, the observation group had lower levels of fasting blood glucose, 2-hour postprandial blood glucose, and glycated hemoglobin than the control group (*P* < .05). Moreover, the observation group had higher visual acuity and lower intraocular pressure than the control group (*P* < .05). The visual impairment rate was lower and the overall compliance rate was higher in the observation group compared to the control group (*P* < .05). After nursing, both groups showed improvements in symptoms, visual function, physical function, psychological and social activity scores, visual acuity, and patient satisfaction scores. The observation group showed greater improvements compared to the control group (*P* < .05). The application of continuous 4C nursing in DR patients has shown positive effects, including improved patient compliance and satisfaction, enhanced patient quality of life and visual acuity. These findings suggest that continuous 4C nursing should be widely promoted and applied in clinical practice.

## 1. Introduction

Diabetic retinopathy (DR) is a group of fundus diseases resulting from chronic progressive diabetes, characterized by leakage and blockage of retinal microvessels. It is a common microvascular complication of diabetes and can significantly impact patients’ vision. If left untreated, it can even lead to blindness, greatly affecting patients’ daily lives and work.^[1–3]^ The trend of DR development is concerning. With the aging population and lifestyle changes in China, the number of diabetic patients is continuously increasing, leading to a rise in DR prevalence. China is one of the countries with the fastest-growing diabetes prevalence globally. It has been reported that the diabetes prevalence among Chinese adults has already exceeded 10%, and it is still on the rise, with an estimated increase to 202.84 million patients by 2050.^[4]^

Existing treatment methods mainly include medication, laser therapy, and surgical intervention. However, these treatments have their limitations. While medication can help control blood sugar and blood pressure, its effectiveness in treating diabetic retinopathy, particularly in advanced stages, is limited. Laser therapy, although it can alleviate retinal edema and bleeding, is not ideal for preserving vision and preventing disease progression and may lead to visual field loss and other side effects. Similarly, surgical intervention, while effective for some complex cases of diabetic retinopathy, carries high risks and recurrence rates.^[5–7]^ Despite significant advancements in medical technology improving visual acuity and quality of life for DR patients posttreatment, the risk of complications such as poor blood sugar control, fundus hemorrhage, and elevated intraocular pressure remains due to a lack of self-care and medication knowledge post-discharge. Therefore, providing effective extended care is crucial to enhance home care effectiveness and improve treatment and rehabilitation levels.^[8,9]^

Continuity of care (4C) nursing, emphasizing comprehensiveness, collaboration, coordination, and continuity, is an outpatient nursing intervention model.^[10]^ It involves guiding patients in blood sugar monitoring, medication management, providing emotional support and psychological counseling to help patients cope with the psychological pressure and anxiety brought about by the disease. Additionally, it includes monitoring changes in the disease condition, early identification, and intervention of signs of disease deterioration, thereby improving treatment effectiveness and prognosis.^[11]^ Therefore, this study primarily explores the application effect of continuous 4C nursing in DR patients and its impact on self-care ability, aiming to provide comprehensive and personalized nursing services to improve patients’ quality of life and reduce the incidence of complications.

## 2. Materials and methods

### 2.1. General data

From October 2020 to October 2022, a total of 100 patients diagnosed with diabetic retinopathy (DR) were recruited from our hospital. Using a random number table method, they were randomly assigned to either a control group or an observation group, with 50 patients in each group. The control group received standard care, while the observation group received continuous 4C care. There were no statistically significant differences in baseline characteristics between the 2 groups (*P* > .05, Supplemental Digital Content, http://links.lww.com/MD/M425). This study was approved by the Ethics Committee of Xinjiang Uygur Autonomous Region People’s Hospital (Approval No. XJWWEZZQRMYY-2020 No. 33), and informed consent forms were signed by the patients and their families.

### 2.2. Method

The 100 study subjects recruited for this research all met the inclusion and exclusion criteria. The inclusion criteria for the study were as follows: meeting the diagnostic criteria for DR^[12]^; undergoing surgical treatment; having type 2 diabetes; and complete preservation of clinical data. The exclusion criteria were as follows: presence of malignant tumors and urinary system diseases; severe dysfunction of important organs such as the heart, kidneys, and liver; complications of other diabetes; presence of neurological disorders, cognitive impairment, and psychiatric disorders with poor treatment compliance; presence of other diseases that affect vision; presence of signs of DR proliferation. Both groups of patients underwent vitrectomy treatment. The control group received routine nursing care, which included providing health education on disease knowledge and hygiene management during the patient’s hospitalization period. Additionally, they paid close attention to the patient’s emotional changes and used a friendly tone to provide psychological counseling. Patients were also guided on how to position themselves properly after surgery, informed about postoperative precautions, and reminded to strictly follow the doctor’s instructions when using eye drops. The control group also received postoperative dietary guidance, where a semiliquid diet was recommended as the main diet for 1 to 2 days after surgery, which could be changed to a regular diet depending on the patient’s condition. The patients were advised to come to the hospital for regular follow-up visits and to seek medical attention promptly if any abnormalities were detected. Furthermore, regular telephone follow-up was conducted to monitor the patients’ recovery status.

The observation group implemented a continuity of 4C care. Comprehensive care was provided by establishing a complete diagnosis and treatment record for the patient during hospitalization. The nursing staff, in conjunction with the supervising nurse and attending physician, comprehensively evaluated the patient’s physical and mental condition, family, and economic situation. They predicted potential physical and mental problems that may arise during the rehabilitation period and provided advanced guidance to the patient and their family on coping strategies. Corresponding nursing intervention models were developed. Patients and their families were informed about the importance of positive emotions in the outcome of the disease and the negative impact of unhealthy lifestyle habits on the disease, aiming to enhance their awareness and improve treatment compliance. Additionally, patients were instructed to press their tongue against the upper palate when coughing to avoid sudden increases in intraocular pressure and wound opening caused by coughing. Comprehensive dietary guidance was provided after surgery, emphasizing a light and easy-to-digest postoperative diet that maintains nutritional balance and avoids smoking and alcohol. Eating small amounts and having multiple meals was recommended to prevent constipation. Patients were advised to eat more bananas and fresh vegetables and, if necessary, given laxatives to avoid redetachment of the retina caused by forced defecation. For patients experiencing severe or frequent vomiting on the day of surgery, eating was paused and metoclopramide was used to stop vomiting. After surgery, it is important to closely monitor the patient’s intraocular pressure. If discomfort symptoms such as headache, nausea, and vomiting persist despite nursing care, it is crucial to inform the doctor immediately. In such cases, the patient’s intraocular pressure should be measured, and appropriate antihypertensive drugs should be administered promptly. Additionally, it is essential to enhance monitoring of intraocular pressure and blood sugar levels. Blood sugar should be measured 6 times before and after each meal on the first day after surgery, and 1 to 3 times a day thereafter. This monitoring helps prevent high or low blood sugar levels from affecting postoperative visual recovery. Patients who have undergone successful surgery and achieved significant treatment outcomes can also be encouraged to share their experiences. This sharing can help reduce negative emotions such as pessimism and anxiety, enabling patients to maintain a positive and optimistic attitude toward their disease and treatment. It can also help them build confidence in the treatment process. Furthermore, providing discharge guidance is crucial. Patients should be educated on how to self-monitor their blood sugar and vision levels, which improves their ability to take care of themselves. For patients requiring visual aids, it is important to instruct them on the correct usage of these instruments to maximize their effectiveness. Patients should also be educated on how to protect their surgical eyes, maintain eye hygiene, and prevent eye infections and complications. Eye drops should be distributed before discharge, and patients and their families should be guided on the correct method of using them to avoid any contact with the surgical eye. Additionally, patients should be advised to sit in the front of the car, fasten their seat belts, and rest firmly on the seat to prevent retinal detachment caused by sudden bumps and vibrations to the head. It is also advisable to minimize riding motorcycles or airplanes if possible. Vigorous exercise, heavy physical labor, and excessive eye use should be avoided for 3 months, and patients should ensure they get sufficient sleep. Collaborative care is essential in ensuring the well-being of patients. It is important to mobilize favorable resources around patients to support their recovery. This can be achieved by strengthening communication and exchange with the patient’s family members before discharge. Providing out-of-hospital health and drug treatment education to the family members helps them understand the role of drug treatment in disease control. Establishing positive cooperation with the family members is crucial. Additionally, it is important to pay attention to the patient’s out-of-hospital rehabilitation stage and actively supervise their medication. Timely feedback on the patient’s out-of-hospital condition to relevant nursing personnel is necessary. Furthermore, providing patients with detailed instructions on postoperative precautions and possible complications is vital. Guiding them on prevention and treatment methods for these complications is important, and if unable to handle them, promptly contacting the hospital to discuss effective solutions together is recommended. Coordinated care involves the collaboration between the responsible nurse and follow-up nurse to establish follow-up files for discharged patients. Regular follow-up work is conducted to gain timely insights into the patient’s psychological status, disease control, daily life, diet, and exercise. This ensures that the patient is informed about and addresses any physical and mental issues that may arise during the recovery period. In case the patient’s condition deteriorates and follow-up nursing is unable to manage it, the responsible nurse should be promptly contacted to arrange for the patient to receive treatment at the hospital. Continuing care: following the patient’s discharge, a weekly telephone follow-up will be conducted to primarily assess their psychological state, daily diet, medication, and exercise, as well as monitor any changes in their condition. In the case of patients experiencing negative emotions, timely lectures on psychological and health knowledge will be provided through the WeChat platform, involving patients, family members, and nursing staff. This aims to offer psychological counseling, correct any misconceptions or unhealthy nursing concepts, and adjust the maintenance plan according to the patient’s specific situation. Additionally, a WeChat group will be created, where patients will be required to report their daily blood sugar levels, blood pressure, and dietary status. The nursing staff will provide targeted guidance based on these reports, answer any questions promptly, and further enhance the patients’ understanding of the disease-related knowledge. This will help improve treatment compliance, prevent unauthorized changes in medication dosage, and ensure patients’ adherence to the prescribed regimen. Moreover, a patient communication group will be established to facilitate communication among patients, allowing them to share disease-related knowledge and self-care experiences. Furthermore, monthly home visits will be conducted to rectify any nonstandard practices, such as blood glucose monitoring and visual acuity assessment, and to ensure medication adherence. Any arising problems will be promptly addressed. Additionally, a monthly health lecture will be organized, inviting renowned experts in the field of DR treatment to educate discharged patients about DR-related knowledge and nursing, guiding them to adopt healthy lifestyle habits. Simultaneously, patients will be guided to implement relaxation measures, such as music therapy and abdominal breathing, at home to alleviate psychological tension. Patients will also be informed about the importance of regular follow-up visits to the hospital, and their follow-up records will be maintained to monitor the progress of the disease. For the loss of follow-up patients due to patient-related factors, changes in contact information, and other personal reasons, we will employ various methods to handle their data, including analyzing the data using the last available observations, conducting sensitivity analysis to assess the impact of lost to follow-up data on the results, among others. In this study, our nursing intervention spans from the first month to the third month after surgery, with postoperative measurements conducted respectively in the first, second, and third months after surgery. Both groups of patients were evaluated for relevant indicators after 2 months of nursing care.

### 2.3. Observation indicators and evaluation criteria

#### 2.3.1. Negative emotions and self-care ability

The self-rating anxiety scale (SAS) was used to assess negative emotions in patients before and after nursing. A score of 50 on the SAS indicates the presence of anxiety, with higher scores indicating more severe negative emotions.^[13,14]^ The exercise of self-care agency scale (ESCA) was used to evaluate a patient’s self-care ability. The ESCA consists of 4 dimensions: health knowledge level, self-responsibility, self-concept, and self-care skills, with a total of 43 items. Each item is scored from 0 to 4 points, resulting in a total score ranging from 0 to 172 points. A higher score on the ESCA indicates better self-care ability.^[15]^

#### 2.3.2. Blood glucose indicators

Fingertip blood samples were collected before and after nursing care, on an empty stomach, and 2 hours after meals, respectively. The Shanghai Abbott optimal blood glucose level was used to measure the levels of fasting blood glucose (FBG) and postprandial blood glucose (C2 h postprandial blood glucose, 2 hours PBG) at 2 hours after meals. Additionally, the Beckman Coulter AU5800, a fully automated biochemical analyzer from the United States, was utilized to detect glycosylated hemoglobin (HbA1c).

#### 2.3.3. Vision, intraocular pressure, and visual impairment

The patient’s vision was measured using an international standard visual acuity chart before and after nursing. Intraocular pressure was measured using the TOPCON CT-80A noncontact tonometer during the same period. The visual acuity decline of patients was calculated, and the evaluation standard was based on a decrease of ≥2 rows in the patient’s original vision.

#### 2.3.4. Comparison of patient compliance

A compliance questionnaire developed by the hospital was utilized to assess patient compliance. The questionnaire covered various aspects such as medication, blood glucose monitoring, diet and exercise, eye care, and regular follow-up. The scale demonstrated good reliability with a coefficient of 0.854 and a Cronbach *α* coefficient of 0.912.

#### 2.3.5. Patient satisfaction comparison

Patient satisfaction was assessed using a self-developed scale, which was subsequently tested for reliability and validity (*α* = 0.812). The scale consisted of a total score of 100 points, with higher scores indicating greater satisfaction.

### 2.4. Statistical analysis

Using SPSS 22.0 statistical software, we represented the quantitative data $\bar{x}\pm s$ and used *t* test for analysis. On the other hand, the count data was represented by *χ*^2^ inspections. A statistically significant difference is indicated when *P* < .05.

## 3. Results

Among the 100 study subjects who met the inclusion and exclusion criteria, there were 50 cases each in the control and observation groups. In the control group, there were 21 males and 29 females, aged between 60 and 79 years, with an average age of (65.26 ± 4.11) years. The average duration of the disease was (6.27 ± 3.33) years, ranging from 2 to 10 years. DR staging in the control group was as follows: stage I, 12 cases; stage II, 30 cases; stage III, 8 cases. In the observation group, there were 22 males and 28 females, aged between 61 and 80 years, with an average age of (65.21 ± 4.24) years. The average duration of the disease was (6.48 ± 2.51) years, ranging from 1 to 10 years. DR staging in the observation group was as follows: stage I, 13 cases; stage II, 31 cases; stage III, 6 cases. There were no statistically significant differences in baseline characteristics between the 2 groups (*P* > .05). Subsequently, statistical software was used to compare negative emotions, self-care ability, blood glucose indicators, postoperative compliance, visual acuity, intraocular pressure, visual impairment, quality of life, and satisfaction before and after nursing in both groups, further exploring the impact of continuous 4C nursing on the self-care ability of DR patients.

### 3.1. Comparison of negative emotions and self-care abilities between 2 groups of patients before and after nursing care

After nursing, the SAS scores of the control group and the observation group decreased from 65.04 ± 5.21 and 65.06 ± 5.18 to 52.11 ± 3.14 and 30.10 ± 3.23, respectively. Conversely, the ESCA scores of the control group and the observation group increased from 70.40 ± 10.13 and 70.38 ± 10.24 to 125.38 ± 10.34 and 160.37 ± 10.30, respectively. Notably, the observation group exhibited significantly greater improvement compared to the control group (*P* < .05, Table 1).

**Table 1:** presents a comparison of negative emotions and self-care ability between the 2 groups of patients before and after nursing, with a sample size of 50 and scores represented as $\bar{x}\pm s$ (points).

Group	SAS score		ESCA score	
	Before	After	Before	After
Control group	65.04 ± 5.21	52.11 ± 3.14*	70.40 ± 10.13	125.38 ± 10.34*
Observation group	65.06 ± 5.18	30.10 ± 3.23*	70.38 ± 10.24	160.37 ± 10.30*
*t*/*P*	0.019/.984	34.549/.000	0.009/.992	4.967/.000

Compared to before nursing.

* *P* < .05.

### 3.2. Comparison of blood glucose indicators between 2 groups of patients before and after nursing care

After nursing, the FBG levels in the control group and observation group were 8.11 ± 2.14 and 5.10 ± 1.23, respectively. The postprandial blood glucose (PBG) levels at 2 hours were 10.01 ± 1.17 and 7.02 ± 1.10, respectively. The HbA1c values were 9.00 ± 1.34 and 7.02 ± 1.30, respectively. All of these values were lower than before nursing, and the observation group had lower values compared to the control group (*P* < .05, Table 2).

**Table 2:** Comparison of blood glucose indicators before and after nursing care between 2 groups of patients (*n* = 50, $\bar{x}\pm s$).

Group	FBG (mmol/L)		2 h PBG (mmol/L)		HhA1c (%)	
	Before	After	Before	After	Before	After
Control group	13.04 ± 2.21	8.11 ± 2.14*	12.20 ± 2.35	10.01 ± 1.17*	10.76 ± 3.13	9.00 ± 1.34*
Observation group	13.15 ± 2.18	5.10 ± 1.23*	12.26 ± 2.30	7.02 ± 1.10*	10.67 ± 3.24	7.02 ± 1.30*
*t*/*P*	0.250/.802	8.622/.000	0.129/.897	13.165/.000	0.141/.887	7.499/.000

Compared to before nursing.

* *P* < .05.

### 3.3. Comparison of postoperative compliance between 2 groups of patients

The compliance rates for postoperative medication, dietary and exercise, eye care, and regular follow-up items in the observation group were 90.00%, 90.00%, 87.50%, and 92.50%, respectively. These rates were higher than those in the control group, and the difference was statistically significant (*P* < .05). Please refer to Table 3 for more details.

**Table 3:** Comparison of postoperative compliance between 2 groups of patients (*n* = 50, %).

Group	Popping pills	Diet and exercise	Eye care	Periodic review
Control group	35 (70.00)	36 (73.50)	34 (67.50)	38 (75.00)
Observation group	45 (90.00)	45 (90.00)	44 (87.50)	46 (92.50)
*t*/*P*	5.000/.025	5.021/.045	4.588/.032	4.501/.034

Compared to before nursing.

*P < .05.

### 3.4. Comparison of visual acuity, intraocular pressure, and visual impairment between 2 groups of patients before and after nursing care

After nursing, the visual acuity of the observation group was 0.91 ± 0.14, which was higher than the control group’s visual acuity of 0.70 ± 0.18. The intraocular pressure of the observation group was 13.28 ± 2.22, which was lower than the control group’s intraocular pressure of 21.59 ± 2.19 (*P* < .05). The visual impairment rate of the observation group was 3/6.00, which was lower than the control group’s rate of 12/24.00. Additionally, the overall compliance rate of the observation group was higher than that of the control group (*P* < .05). Please refer to Table 4 for more details.

**Table 4:** Comparison of visual acuity, intraocular pressure, and visual impairment between 2 groups of patients before and after nursing care (*n* = 50).

Group	Vision (*x* ± *s*)		Intraocular pressure (*x* ± *s*, mm Hg)		Visual impairment (*n*%)
	Before	After	Before	After	
Control group	0.41 ± 0.11	0.70 ± 0.18*	26.75 ± 2.48	21.59 ± 2.19*	12/24.00
Observation group	0.42 ± 0.13	0.91 ± 0.14*	26.93 ± 2.27	13.28 ± 2.22*	3/6.00
*t*/*P*	0.415/.678	6.511/.000	0.378/.705	18.843/.000	6.352/.011

Compared to before nursing.

* *P* < .05.

### 3.5. Comparison of quality of life between 2 groups of patients

Prior to nursing intervention, there was no statistically significant difference in SQQL-VI scores between the 2 groups (*P* > .05). However, after nursing intervention, both groups showed higher scores compared to before nursing, and the observation group had significantly higher scores than the control group (*P* < .05). Please refer to Table 5 for further details.

**Table 5:** Comparison of quality of life between 2 groups of patients (*n* = 50, $\bar{x}\pm s$, points).

Group	Physical functions		Spiritual psychology		Social activities	
	Before	After	Before	After	Before	After
Control group	42.31 ± 5.97	60.89 ± 7.91*	36.58 ± 5.05	47.81 ± 6.25*	34.27 ± 4.52	46.13 ± 6.15*
Observation group	42.06 ± 5.95	65.43 ± 8.65*	36.24 ± 5.03	51.63 ± 6.90*	34.38 ± 4.54	49.42 ± 7.80*
*t*/*P*	0.186/.852	2.450/.017	0.302/.764	2.595/.011	0.109/.914	2.095/.039

Compared to before nursing.

* *P* < .05.

### 3.6. Comparison of satisfaction between 2 groups

Prior to nursing intervention, there was no statistically significant difference in visual acuity and patient satisfaction scores between the 2 groups (*P* > .05). However, after nursing intervention, the satisfaction scores of the observation group and the control group improved to 90.32 ± 9.54 and 85.78 ± 8.01, respectively. These scores were higher compared to the scores before nursing intervention, which were 75.20 ± 6.95 and 75.12 ± 6.93, respectively. Furthermore, the observation group had a higher satisfaction score than the control group, and this difference was statistically significant (*P* < .05). Please refer to Table 6 for further details.

**Table 6:** Comparison of satisfaction between 2 groups (*n* = 50, $\bar{x}\pm s$).

Group	Satisfaction	
	Before	After
Control group	75.12 ± 6.93	85.78 ± 8.01*
Observation group	75.20 ± 6.95	90.32 ± 9.54*
*t*/*P*	0.052/.959	2.305/.024

Compared to before nursing.

* *P* < .05.

## 4. Discussion

DR is primarily caused by damage to retinal microvessels, with a high incidence rate. Currently, Chinese diabetic patients do not pay sufficient attention to retinal lesions, leading to prolonged hyperglycemia, damage to retinal microvessels, and even blindness in severe cases.^[16]^ In contrast to previous studies that mainly focused on single-treatment modalities such as medication, laser therapy, or surgical treatment, this study explored a comprehensive intervention approach through continuous 4C nursing, which provides a more holistic care plan for DR patients.^[5,7]^ While traditional nursing interventions primarily occur during hospitalization, posthospital care interventions are often overlooked, limiting patients’ self-care abilities after discharge and resulting in suboptimal nursing outcomes.^[17,18]^ Therefore, there is a need to further explore more suitable nursing intervention methods to meet specific requirements.

In this study, the innovative continuous 4C nursing model was introduced, emphasizing continuous nursing interventions outside the hospital, which is in contrast to traditional inpatient care methods. Through this innovative nursing model, a new perspective was provided to explore the nursing needs of DR patients. Additionally, unlike previous studies that mainly focused on specific patient outcomes such as visual recovery or complication rates, this study provided a more comprehensive assessment of results by evaluating multiple aspects such as self-care ability, negative emotions, blood glucose control, and postoperative compliance. The core principle of continuity nursing based on the 4C model played a crucial role in the entire nursing intervention process across various departments, yielding favorable outcomes.^[19]^ Compared to traditional nursing models, the 4C-based continuity nursing model established seamless connections between responsible nurses, follow-up nurses, patients, and family members. Comprehensive patient assessments were conducted, and health records were established, followed by the formulation of appropriate nursing intervention plans. After discharge, patients were guided in self-care, while family members and follow-up nurses provided supervision and support, ensuring the continuity and effectiveness of postoperative care. In this study, the compliance of postoperative indicators (medication, blood glucose monitoring, diet and exercise, eye care, and regular follow-up) in the observation group was significantly higher than that in the control group (*P* < .05), consistent with previous research findings. The 4C model-based continuity nursing significantly improved patient compliance, resulting in better postoperative quality of life for DR patients.^[20,21]^ The observation group exhibited higher postoperative quality of life indicators, including symptoms and visual function, physical function, psychological and social activity, compared to the control group, with statistical significance (*P* < .05). Moreover, the observation group showed significantly higher visual acuity and patient satisfaction scores than the control group (*P* < .05), indicating better visual acuity and patient recognition with continuity nursing based on the 4C model.

These results highlight the importance of continuity nursing based on the 4C model in improving daily quality of life and gaining patient recognition, consistent with the continuity nursing theory proposed by Boucher et al.^[22,23]^ Previous studies by Boucher Marie Carole et al have also demonstrated that nursing models can improve patient compliance, enhance patient quality of life, and ultimately improve patient recovery after surgery.^[23,24]^

This study fills knowledge gaps in the existing literature and provides an important theoretical and practical foundation for further research in this field. It is of significant importance for improving patient treatment outcomes and quality of life, reducing complication rates, and enhancing overall patient health. Despite achieving some positive results, the study has limitations such as a relatively small sample size, short study duration, and single-center design, which may affect the generalizability and reliability of the results. Future research could address these limitations by expanding the sample size, extending the study duration, and engaging in multicenter collaborations.

## Author contributions

**Conceptualization:** Ayixianmuguli Wufuer, Jiamei Ma, Pazilaiti Ainiwa, Qi Zhou.

**Data curation:** Ayixianmuguli Wufuer, Jiamei Ma, Pazilaiti Ainiwa, Qi Zhou.

**Formal analysis:** Ayixianmuguli Wufuer, Jiamei Ma, Pazilaiti Ainiwa, Qi Zhou.

**Funding acquisition:** Ayixianmuguli Wufuer, Pazilaiti Ainiwa, Qi Zhou.

**Investigation:** Ayixianmuguli Wufuer, Pazilaiti Ainiwa.

**Writing – original draft:** Ayixianmuguli Wufuer, Jiamei Ma.

**Writing – review & editing:** Ayixianmuguli Wufuer, Jiamei Ma, Pazilaiti Ainiwa.

**Supervision:** Jiamei Ma, Qi Zhou.

## Supplementary Material
